# Correction: The effect of dietary interventions on inflammatory biomarkers among people with multiple sclerosis: A protocol for systematic review and meta-analysis of randomized controlled trials

**DOI:** 10.1371/journal.pone.0334893

**Published:** 2025-10-16

**Authors:** Farnoosh Shemirani, Wade R. Pingel, Tyler J. Titcomb, Asma Salari-Moghaddam, Farshad Arsalandeh, Solange M. Saxby, Linda G. Snetselaar, Terry L. Wahls

There is an error in affiliation 4 for author Farshad Arsalandeh. The correct affiliation 4 is: Department of Neuroscience, Faculty of Advanced Technologies in Medicine, Iran University of Medical Sciences, Tehran, Iran.
