# Supplementary material for: The effect of dietary interventions on inflammatory biomarkers among people with multiple sclerosis: A protocol for systematic review and meta-analysis of randomized controlled trials
Source: PLoS One. 2024 Feb 7;19(2):e0297510. doi: 10.1371/journal.pone.0297510 (PMC10849228; doi:10.1371/journal.pone.0297510)
Supplement: S1 File — (PDF) [file pone.0297510.s002.pdf]

Search Syntax for PubMed/MEDLINE

(“diet”[Mesh] OR “diet therapy”[Mesh] OR “nutrition therapy”[Mesh] OR diet[all] OR dietary[all] OR nutrition[all] OR nutritional[all] OR Paleolithic[all] OR Paleo[all] OR “modified paleolithic diet”[all] OR “elimination diet”[all] OR “High-Fat Diet”[all] OR high protein[all]OR fat restricted[all] OR low fat[all] OR Ketogenic[all] OR “modified ketogenic diet”[all] OR keto[all] OR ketogenous[all] OR ketotic[all] OR Atkins[all] OR FODMAP[all] OR DASH[all] OR MIND[all] OR Gluten Free[all] OR “Low Carbohydrate”[all] OR “Carbohydrate Restricted”[all] OR Mediterranean[all] OR “modified Mediterranean”[all] OR Vegetarian[all] OR vegan[all] OR plant based[all] OR “Lacto Vegetarian”[all] OR low glycemic[all] OR low sodium[all] OR fasting[all] OR FMD[all] OR “Time Restricted Eating”[all] OR “time restricted feeding”[all] OR “Calorie Restriction”[all] OR “low calorie”[all] OR “control diet”[all] OR “usual diet”[all] OR “standard diet”[all] OR “anti-inflammatory diet”[all]) AND ("intercellular signaling peptides and proteins"[Mesh] OR "inflammatory markers"[all] OR cytokine[all] OR adipokine[all] OR adipocytokine[all] OR "Nuclear Factor-Kappab"[all] OR "Transcription Factor NF-kB"[all] OR "NF-kB"[all] OR "C Reactive Protein"[all] OR hsCRP[all] OR "High Sensitivity C-Reactive Protein"[all] OR hs-CRP[all] OR interleukin[all] OR IL[all] OR “Tumor Necrosis Factor alpha”[all] OR TNF[all] OR “Transforming Growth Factor”[all] OR TGF[all] OR ‶Intercellular Adhesion Molecule”[all] OR ICAM[all] OR leptin[all] OR adiponectin[all] OR biomarker[all]) AND ("Multiple Sclerosis"[Mesh] OR multiple sclerosis[all] OR disseminated sclerosis[all])
