# Supplementary material for: The effect of dietary interventions on inflammatory biomarkers among people with multiple sclerosis: A protocol for systematic review and meta-analysis of randomized controlled trials
Source: PLoS One. 2024 Feb 7;19(2):e0297510. doi: 10.1371/journal.pone.0297510 (PMC10849228; doi:10.1371/journal.pone.0297510)
Supplement: S1 Table — (PDF) [file pone.0297510.s003.pdf]

Table 1. Main characteristics of RCTs examining the effects of dietary patterns on inflammatory biomarkers in adults with MS.

First author (Year)	Country	No. of Participants	Age range	Design	intervention	Control	Duration	Outcome	
								intervention	Control
