# Supplementary material for: The effect of dietary interventions on inflammatory biomarkers among people with multiple sclerosis: A protocol for systematic review and meta-analysis of randomized controlled trials
Source: PLoS One. 2024 Feb 7;19(2):e0297510. doi: 10.1371/journal.pone.0297510 (PMC10849228; doi:10.1371/journal.pone.0297510)
Supplement: Attachment — Submitted filename: Response to Reviewers.docx [file pone.0297510.s004.docx]

**Responses to Reviewers' comments**

The authors express their gratitude to all the reviewers for providing valuable comments and dedicating efforts to enhance our manuscript. Diligent attention has been given to refining the work and addressing their concerns. All requested journal requirements have been addressed and included either in the revised manuscript or within the cover letter. The following are the point-by-point responses to the reviewers’ comments/questions.

Reviewers' comments:
Reviewer's Responses to Questions

**Comments to the Author**

1. Does the manuscript provide a valid rationale for the proposed study, with clearly identified and justified research questions?

The research question outlined is expected to address a valid academic problem or topic and contribute to the base of knowledge in the field.

Reviewer #1: Yes

Reviewer #2: Yes

**Reviewer #3: Partly**

2. Is the protocol technically sound and planned in a manner that will lead to a meaningful outcome and allow testing the stated hypotheses?

The manuscript should describe the methods in sufficient detail to prevent undisclosed flexibility in the experimental procedure or analysis pipeline, including sufficient outcome-neutral conditions (e.g. necessary controls, absence of floor or ceiling effects) to test the proposed hypotheses and a statistical power analysis where applicable. As there may be aspects of the methodology and analysis which can only be refined once the work is undertaken, authors should outline potential assumptions and explicitly describe what aspects of the proposed analyses, if any, are exploratory.

Reviewer #1: Yes

Reviewer #2: Yes

Reviewer #3: Yes

3. Is the methodology feasible and described in sufficient detail to allow the work to be replicable?

Descriptions of methods and materials in the protocol should be reported in sufficient detail for another researcher to reproduce all experiments and analyses. The protocol should describe the appropriate controls, sample size calculations, and replication needed to ensure that the data are robust and reproducible.

**Reviewer #1: No**

Reviewer #2: Yes

Reviewer #3: Yes

4. Have the authors described where all data underlying the findings will be made available when the study is complete?
The [PLOS Data policy](https://journals.plos.org/plosone/s/materials-and-software-sharing) requires authors to make all data underlying the findings described in their manuscript fully available without restriction, with rare exception, at the time of publication. The data should be provided as part of the manuscript or its supporting information, or deposited to a public repository. For example, in addition to summary statistics, the data points behind means, medians and variance measures should be available. If there are restrictions on publicly sharing data—e.g. participant privacy or use of data from a third party—those must be specified.

Reviewer #1: Yes

Reviewer #2: Yes

Reviewer #3: Yes

5. Is the manuscript presented in an intelligible fashion and written in standard English?
PLOS ONE does not copyedit accepted manuscripts, so the language in submitted articles must be clear, correct, and unambiguous. Any typographical or grammatical errors should be corrected at revision, so please note any specific errors here.

Reviewer #1: Yes

Reviewer #2: Yes

Reviewer #3: Yes

Reviewer #1:

**Q1**: The study would benefit from a special trigger on known cross-reactivity of myelin-related proteins and dietary proteins.

**A1**: We appreciate the valuable suggestion from the reviewer. Exploring the interplay between myelin-related proteins and dietary proteins is an intriguing topic that could be considered for investigation in a separate systematic review. The present protocol for systematic review and meta-analysis is focused on examining the potential impacts of dietary patterns, as opposed to individual dietary components, on alterations in serum/plasma levels of inflammatory biomarkers, including adipokines and cytokines.

**Q2**: Inclusion of the following references in the introduction may also benefit the work for comprehensiveness:
<https://doi.org/10.1093/intimm/dxaa054>
<https://doi.org/10.1016/j.jtauto.2019.100003>
<https://doi.org/10.1016/j.autrev.2020.102459>

A2: We appreciate the reviewer for offering the inclusion of the suggested references. The three references primarily delve into the dietary antigens of specific food components such as pork, milk, and cheese. They explore the potential correlation between these components and autoimmunity, discussing topics like dietary-human autoimmune epitope overlap, immunogenetics, and the potential autoimmunogenicity of certain foods. To enhance the comprehensiveness of the manuscript, we have incorporated the second reference (<https://doi.org/10.1016/j.jtauto.2019.100003>) into the introduction section (line 84, reference 18).

**Q3**. Is the methodology feasible and described in sufficient detail to allow the work to be replicable?

Descriptions of methods and materials in the protocol should be reported in sufficient detail for another researcher to reproduce all experiments and analyses. The protocol should describe the appropriate controls, sample size calculations, and replication needed to ensure that the data are robust and reproducible.

**Reviewer #1: No**

A3: In addressing the reviewer's concern about the feasibility and clarity of the methodology for potential replication, we want to highlight that the search syntax employed in the PubMed database is available in S2 Table, aiding in the replication of our study's findings. Furthermore, a comprehensive methodological description, encompassing eligibility criteria, study design, search strategy, study selection, data extraction, and credibility assessment of evidence, can be found in Section 2 and its subheadings (2.1 – 2.5).

Reviewer #2:

The Effect of Dietary Interventions on Inflammatory Biomarkers Among People with Multiple Sclerosis: A Protocol for Systematic Review and Meta-Analysis of Randomized Controlled Trials.

The article/protocol is very good and useful for specialists who want to perform the systematic review and meta-analysis of randomized controlled trials regarding the effect of dietary interventions on inflammatory biomarkers among people with multiple sclerosis.
In addition, systematic reviews and meta-analyses of clinical trials concerning these aspects in MS are lacking. Thus, I agree with the publication of this study protocol. Moreover, the manuscript is well-structured and comprehensible.

**A3**: We appreciate the reviewer's positive feedback on our protocol. We are grateful for their recognition of the manuscript's structure and clarity, as well as for supporting the publication of our study protocol.

Reviewer #3:

The authors aim to evaluate the effects of dietary interventions on inflammatory markers in adults with MS.

Comments:
Introduction: lines 56-57: "The induction of inflammation by the immune system is linked with several factors, including adipose tissue-associated cytokine and adipokine production (7, 8)."

**Q4**: From this sentence it seems like adipokines are the main mechanism involved in the MS pathophysiology. The induction of inflammation could be genetically provoked in genetically susceptible individuals, by microorganisms, by hypovitaminoses D, etc. Please insert more explanations and additional references.

**A4**: We thank the reviewer for making this comment. We have added additional explanation and cited more relevant references for more clarity. Please see lines 47-50, and 66-73 in the revised manuscript.

**Q5**: If applicable, we recommend that you deposit your laboratory protocols in protocols.io to enhance the reproducibility of your results. Protocols.io assigns your protocol its own identifier (DOI) so that it can be cited independently in the future. For instructions see: <https://journals.plos.org/plosone/s/submission-guidelines#loc-laboratory-protocols>. Additionally, PLOS ONE offers an option for publishing peer-reviewed Lab Protocol articles, which describe protocols hosted on protocols.io. Read more information on sharing protocols at <https://plos.org/protocols?utm_medium=editorial-email&utm_source=authorletters&utm_campaign=protocols>.

**A5:** Thank you for your suggestion. It's important to note that the present manuscript serves as a protocol for a systematic review and meta-analysis, categorized as a Study Protocol. We have adhered to the reporting guidelines specific to protocols, namely PRISMA-P, as outlined in the "Guidelines for Specific Study Types", and have already registered the study protocol in PROSPERO International Prospective Register of Systematic Reviews (registration number: CRD42023425961). We will make sure to choose the correct study type when submitting the revision.

**Q6**: Does the manuscript provide a valid rationale for the proposed study, with clearly identified and justified research questions?

The research question outlined is expected to address a valid academic problem or topic and contribute to the base of knowledge in the field.

**Reviewer #3: Partly**

**A6**: We appreciate the reviewer for raising this concern. in response, we would like to highlight the notion that our research rationale stems from the gaps observed in the current literature. Clinically, the course of MS has been associated with inflammatory mediators including cytokines such as IL-1β, IL-6, IL-17, TNF-α, and INF-γ. The extensive body of research on inflammation in MS has laid a foundation, pointing towards the potential therapeutic role of diet. Because many pharmacological treatments have significant limitations, individuals with MS seek non-pharmacologic therapies to mitigate fatigue and reduce their symptoms. Clinical trials and animal studies support that diet profoundly impacts MS severity and disease trajectory. Yet, evidence for the mechanism of action of therapeutic diets, as opposed to individual dietary components, remains inconsistent.

Given this inconsistency and the limited role of neurologists in providing dietary recommendations, newly diagnosed people with MS typically receive minimal dietary guidance. In turn, many individuals seek dietary information from internet sources, which are not often evidence-based. Thus, the specific influence of dietary patterns on inflammatory markers lacks comprehensive exploration, and a systematic review is crucial to consolidate the existing evidence.

Providing further evidence on the link between dietary interventions and inflammation within the MS community stands to benefit not only individuals dealing with MS but also their healthcare providers, support persons, and families. It's crucial to provide well-informed, evidence-based dietary guidance to address a significant gap in current MS management practices. The last paragraph of the introduction (lines 95-105) has been revised to articulate the study's rationale more clearly.
